# Peripheral-central inflammatory crosstalk in Alzheimer’s disease: immunometabolic functions of circulating soluble metabolites under dynamic blood-brain barrier states

**DOI:** 10.3389/fimmu.2026.1939482

**Published:** 2026-08-28

**Authors:** Nannan Song, Huan Xing, Feng Liu, Wang Xi

**Affiliations:** Jiangxi University of Chinese Medicine (JXUCM), Nanchang, China

**Keywords:** Alzheimer’s disease, blood-brain barrier, circulating soluble metabolites, immunometabolism, neuroinflammation, peripheral-central inflammatory crosstalk

## Abstract

Immunometabolic dysregulation orchestrating bidirectional peripheral–central inflammatory crosstalk has been increasingly recognized as a contributor to Alzheimer’s disease (AD) pathogenesis, challenging the conventional view of AD as an isolated central disorder dominated by amyloid-beta (Aβ) and tau pathology. However, although central immune-metabolic disturbances are well established, this perspective largely overlooks peripherally derived regulatory signals from systemic circulation. Classical peripheral-to-central pathways, such as immune cell infiltration and cytokine leakage, depend on overt blood–brain barrier (BBB) damage and cannot account for the latent neuroinflammation characteristic of the prodromal stage, when neurovascular units remain largely intact. To address this gap, we review recent advances on circulating soluble metabolites (<500 Da)—key immunometabolic mediators linking systemic disturbances to central lesions—and organize them into three groups by biosynthetic origin (a heuristic, non-taxonomic classification): peripheral immune-derived, gut microbiota-derived, and systemic host metabolic intermediates. We outline the transmembrane transport shifts associated with progressive BBB dysfunction and the downstream intracellular cascades—such as mitochondrial redox disturbance, mTOR–NLRP3 inflammasome activation, and histone epigenetic remodeling—that mediate this crosstalk. Importantly, although these proposed associations are mechanistically informative, they remain largely unsupported by direct human evidence and require further validation in large AD cohorts; nevertheless, several individual metabolite biomarkers already show consistent clinical association. This framework clarifies the multi-stage immunometabolic axis and may inform the development of ultra-early fluid biomarkers and peripheral-targeted immunometabolic interventions.

## 1 Introduction

Immunometabolic dysregulation has been increasingly recognized as a contributor to persistent pathological neuroinflammation—a feature implicated in Alzheimer’s disease (AD) progression (1). Early AD research adopted a brain-centric perspective, classifying AD as an isolated central neurodegenerative disorder dominated by cerebral amyloid-beta (Aβ) deposition and tau hyperphosphorylation, while overlooking the upstream regulatory effects of peripheral immune and metabolic imbalance (2). Recent multi-omics and gut-brain axis cohort data inform a broader systemic conceptualization of AD, in which chronic peripheral low-grade inflammation is proposed to transmit pathological signals across the neurovascular unit and trigger latent preclinical central immune dysfunction (3, 4).

Multiple canonical pathways mediate peripheral inflammatory signal transmission to the central nervous system (CNS), including immune cell infiltration, macromolecular cytokine transcytosis, and exosome cargo delivery (5). Nevertheless, these routes have largely been characterized in the context of mid-to-late AD, whereas the relative contribution of soluble metabolites at the prodromal stage remains to be established, failing to explain subtle low-grade neuroinflammation when neurovascular units (NVUs) remain structurally intact (6). Circumventricular organs (CVOs) penetration and vagal afferent signaling are anatomically restricted to limited brain regions and cannot relay systemic pathological signals to the broader brain parenchyma (7). This creates a critical research gap: which circulating molecular mediators connect systemic immunometabolic disturbance to early central neuronal injury under intact BBB conditions?

Circulating soluble metabolites with a molecular weight below 500 Da serve as a stable molecular bridge between peripheral and central compartments, linking systemic immunometabolic disturbances to central pathology. For clarity of exposition, these bioactive molecules are organized—as a conceptual framework rather than a strict biological taxonomy—by their principal biosynthetic origin into three subgroups, each examined in turn below: (i) peripheral immune-derived mediators, released or reprogrammed by activated immune cells; (ii) gut microbiota-derived regulatory metabolites, generated through microbial fermentation and host biotransformation; and (iii) systemic host metabolic intermediates, arising from endogenous host metabolism. Although individual metabolites within each category have been investigated in depth, an integrated framework that reconciles their stage-dependent trans-barrier transport, convergent intracellular signaling, and clinical translational value has remained lacking.

To fill this gap, the present review elaborates on the biosynthetic sources, differential transport features, and bidirectional immunomodulatory phenotypes of the three metabolite subgroups. We further dissect the sequential intracellular inflammatory cascades induced after metabolites infiltrate brain tissue, including mitochondrial redox disorder, mTOR-NLRP3 inflammasome activation, and histone epigenetic remodeling. Finally, this work summarizes translational prospects for these metabolites as ultra-early fluid biomarkers and peripheral immunometabolic intervention targets, objectively evaluates existing research limitations, and proposes future research directions. This integrated immunometabolic framework offers an integrative perspective on the peripheral-origin pathogenesis of AD and may inform future theoretical development for early diagnosis and targeted therapy.

## 2 AD peripheral-central inflammatory crosstalk: trans-barrier mechanisms and gaps

### 2.1 Progressive functional impairment of the BBB in AD pathological progression

The blood-brain barrier (BBB) maintains central neuroimmune and metabolic homeostasis and establishes CNS immune privilege through physical isolation and selective transmembrane transport (8). The integrated barrier system comprises the canonical BBB, the blood-cerebrospinal fluid barrier, the glymphatic system and meningeal lymphatic vessels. Neurovascular units (NVUs)—formed by endothelial cells, pericytes and astrocytic endfeet—serve as its functional core (9).

During prodromal AD and mild cognitive impairment (MCI), the tight-junction architecture remains largely intact. Nevertheless, accumulated amyloid-beta (Aβ) oligomers and hyperphosphorylated tau trigger pericyte loss and astrocyte depolarization, which downregulate tight-junction proteins and enhance endothelial transcytosis (10). This mild defect does not permit large-scale immune-cell infiltration, yet it compromises the selective filtration of circulating small metabolites, allowing peripheral toxic substances to penetrate and disrupt central immune tolerance.

In mid-stage AD, tight-junction expression declines further and mild paracellular gaps emerge, permitting limited leakage of inflammatory macromolecules. In late AD, irreversible degradation of tight-junction complexes creates persistent endothelial gaps that enable unrestricted infiltration of peripheral immune cells and inflammatory factors, thereby establishing a vicious cycle of glial overactivation and sustained BBB dysfunction (8). This three-stage deterioration explains stage-specific differences in peripheral inflammatory signal transmission across the AD spectrum.

### 2.2 Canonical pathways of peripheral-central inflammatory crosstalk in AD

**Figure 1:**
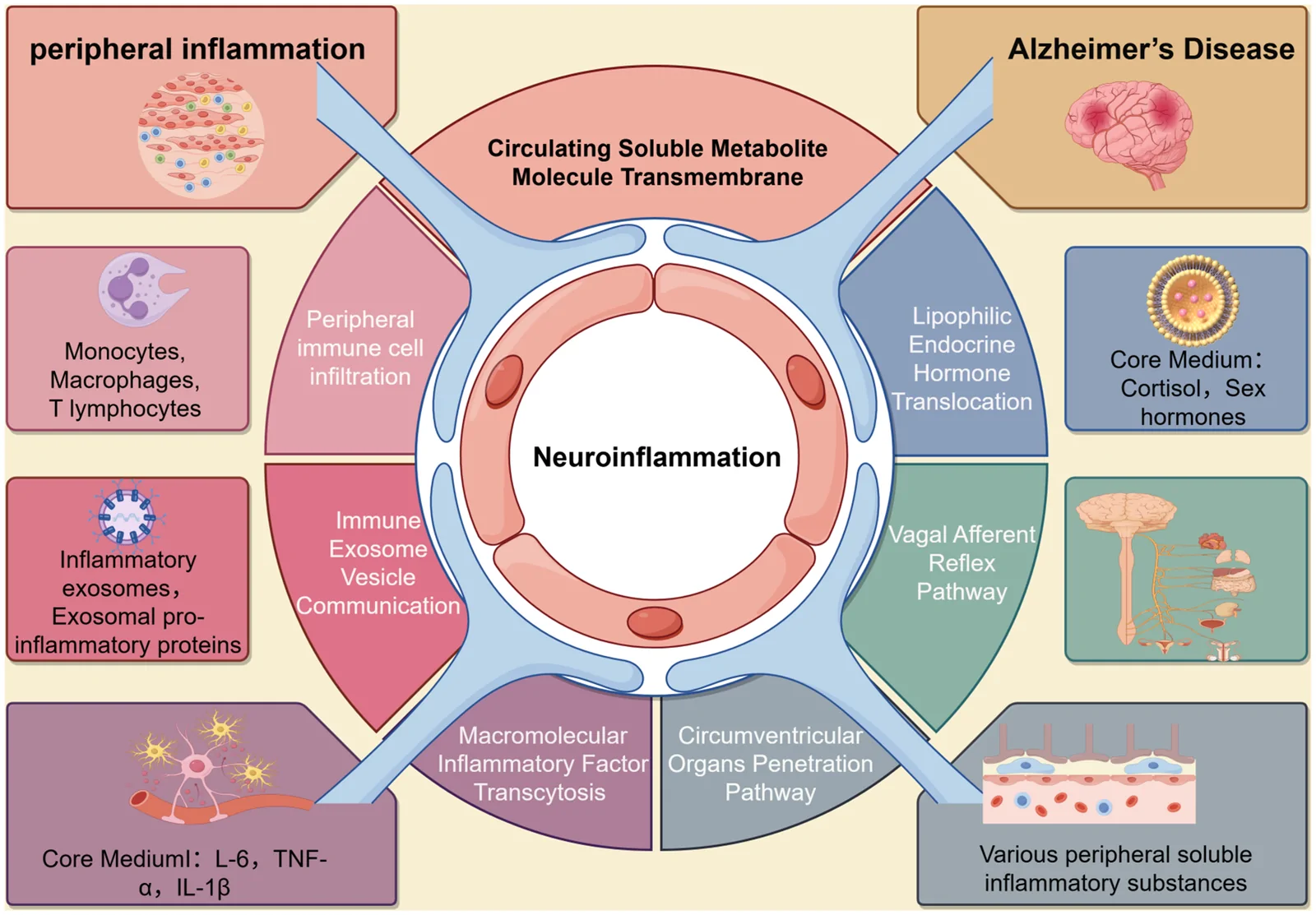
Seven peripheral inflammatory signaling pathways across the intact and damaged blood-brain barrier in Alzheimer’s disease. This schematic summarizes seven major pathways by which peripheral inflammatory mediators cross the blood-brain barrier (BBB) and trigger central pathological lesions in Alzheimer’s disease (AD). These pathways differ in transport carriers, anatomical characteristics and functional stages of AD progression. Created by figdraw.com.

As a highly selective partition separating the systemic circulation from the CNS, the BBB fails to fully preclude peripheral inflammatory signaling. Seven established transmission routes with distinct anatomical constraints and stage-specific adaptabilities are summarized below (**Figure 1**):

1. Peripheral immune cell infiltration:​ Monocytes, macrophages, neutrophils, and CD4^+^/CD8^+^ T lymphocytes act as cellular inflammatory effectors. Their transendothelial extravasation principally relies on structural tight junction damage and endothelial retraction (11). Upon infiltrating brain parenchyma, these cells secrete high levels of IL-1β, TNF-α, and IFN-γ. On the available evidence, this pathway appears to contribute prominently to inflammatory amplification in mid-to-late AD and preferentially targets brain regions enriched in tau tangles over those with Aβ plaques.
2. Macromolecular cytokine transcytosis and paracellular leakage:​ Pro-inflammatory macromolecules such as IL-6 and TNF-α exhibit biphasic transport characteristics. When the BBB remains intact, only trace cytokines are delivered via limited receptor-mediated transcytosis to generate subtle inflammatory cues (12). Once tight junctions disintegrate, paracellular gaps permit massive, non-selective cytokine leakage into the brain parenchyma.
3. Circumventricular organ (CVO) penetration:​ The subfornical organ and vascular organ of the lamina terminalis lack continuous endothelial tight junctions, enabling free diffusion of circulating inflammatory mediators. However, CVOs are anatomically confined to the hypothalamus and brainstem, rendering them incapable of relaying inflammatory signals to the broader brain (13).
4. Lipid-soluble endocrine hormone modulation:​ Lipophilic hormones—including cortisol, estrogen, and androgen—cross the intact BBB via passive diffusion and exert immunomodulatory effects through the hypothalamic-pituitary-adrenal (HPA) axis (14). Chronic stress-induced hormonal dysregulation only mildly activates microglia and functions as a secondary modulator rather than a primary pro-inflammatory stimulus.
5. Vagal afferent reflex signaling:​ The vagus nerve transmits peripheral visceral inflammatory signals to the nucleus tractus solitarius in the brainstem via electrical impulses, independent of circulating humoral factors (15). This rapid reflex pathway predominantly regulates subcortical regions and lacks whole-brain modulatory capacity.
6. Immune exosome vesicle communication:​ Peripheral exosomes laden with inflammatory mRNA, microRNA, and proteins enter the CNS via endothelial endocytosis (16). Nevertheless, individual exosomes carry limited inflammatory cargo, resulting in constrained aggregate pro-inflammatory potential.
7. Circulating soluble metabolite transport:​ Notably, low-molecular-weight metabolites (<500 Da)—such as lactate and short-chain fatty acids—traverse the BBB via carrier-mediated transport and passive diffusion without requiring tight junction disruption. Unlike the aforementioned six pathways—which either depend on late-stage barrier rupture or are restricted to discrete brain regions—soluble metabolites can deliver inflammatory signals across the entire brain even during the prodromal phase when the BBB is functionally intact.

### 2.3 Immuno-metabolic mediators: gateway to central pathology behind an intact BBB

Among the seven trans-BBB signal transmission pathways, circulating soluble metabolites possess unique transport patterns and immunomodulatory properties that cannot be replicated by other peripheral inflammatory carriers. Different from immune cells and protein cytokines that require BBB rupture or partial anatomical confinement, these bioactive small molecules access the CNS through dual transport modes without tight junction disruption, achieving uniform inflammatory signal transmission across the whole brain in prodromal AD (17).

More importantly, core metabolites such as lactate and succinate have been proposed to act not only as transient triggers but also as epigenetic modifiers that may durably influence the transcriptional landscape of glia and neurons, whereas immune cells and cytokines are generally associated with more transient responses (18). This distinctive mechanistic feature distinguishes metabolite-mediated crosstalk from other peripheral signal pathways and may account for its research and translational interest. It extends—rather than replaces—the conventional research framework centered on cellular infiltration and macromolecular leakage, offers a complementary perspective for explaining the origin of silent early neuroinflammation, and highlights candidate molecules for developing non-invasive early diagnostic markers and peripheral immunoregulatory therapies targeting AD; we emphasize that these proposed links remain to be validated in human cohorts.

## 3 Classification system and core characteristics of circulating metabolic small molecules

Circulating soluble metabolites, despite their chemical heterogeneity, share the capacity to initiate or modulate stage-specific pathogenic cascades across an anatomically intact blood–brain barrier (BBB). To impose order on this diverse signaling space, the metabolites examined herein are grouped by their dominant biosynthetic origin into three principal categories: (i) peripheral immune-derived mediators, released or reprogrammed by activated immune cells; (ii) gut microbiota-derived regulatory metabolites, generated through microbial fermentation and host biotransformation; and (iii) systemic host metabolic intermediates, arising from endogenous host metabolism. This tripartite division is adopted as a heuristic organizational framework rather than a rigid biological taxonomy, and its scope and representative members are delineated in the subsections that follow. To facilitate critical appraisal, the level of evidence supporting each metabolite—spanning cell culture, animal models, clinical cohorts, and systematic reviews—is annotated in the Evidence Level columns of **Tables 1**–**3**; the majority of mechanistic links presently rest on preclinical data and await validation in human AD cohorts.

**Table 1:** Peripheral immune-derived soluble metabolic small molecules.

Molecule	MW(Da)	Generation source	BBB crossing mechanism	Central pathogenic/protective mechanism	Evidence level
Lactate	90.08	Peripheral immune cell glycolysis (Warburg effect), TLR4/NF-κB activation	MCT1/2/4 mediated active transport	Histone lactylation (H4K12la/H3K18la) → NF-κB activation; APP K612/tau K331 lactylation; Epigenetic memory	Animal + *In vitro* (19, 20)
Succinate	​ 118.09	Immune cell SDH dysfunction + Reverse Electron Transfer (RET), TCA cycle obstruction	OAT/OATP mediated transport/Passive diffusion	UCNR1→HIF-1α/ROS axis; GSK-3β activation → tau hyperphosphorylation	Animal + *In vitro* (21, 22)
Itaconate	130.07	Immune cell IRG1 catalyzes cis-aconitate decarboxylation	Passive diffusion	Competitive inhibition of SDH → block RET; Nrf2 activation → suppress NLRP3 priming	Animal + *In vitro* (23, 24)
Kynurenic Acid	189.17	Protective branch of tryptophan metabolism, KAT enzyme catalysis	LAT1 (SLC7A5) mediated transport	Antagonize NMDA/α7nAChR; Promote microglial M2 polarization	Human + Animal + *In vitro* (25–27)
α-ketoglutarate	146.10 Da	Activated monocytes, macrophages, T cells (TCA cycle intermediate accumulated during immune metabolic reprogramming)	Organic anion transporters	Remodel microglial epigenetics, increase ROS, cooperate with lactate and succinate to worsen tau phosphorylation and synaptic injury	Human + Animal + *In vitro* (28–30)
Citrate	192.12 Da	Activated myeloid immune cells (massively secreted upon glycolytic shift)	Organic anion transporters	Break microglial redox balance, strengthen neuroinflammation induced by core immune metabolites	Animal + *In vitro* (23, 29)

*Evidence Level: Human/Clinical = evidence derived from human/clinical studies (e.g., patient cohorts, CSF/serum metabolomics); Animal = in vivo animal models; In vitro = cell-culture experiments. ‘+’ indicates combined evidence across multiple levels.

**Table 2:** Gut microbiota-derived soluble metabolic small molecules.

Molecule	MW(Da)	Generation source	BBB crossing mechanism	Central pathogenic/protective mechanism	Evidence level
Acetate	60.05	Commensal fermentation of dietary fiber	MCT mediated active transport	Inhibit peripheral glycolysis; Upregulate BBB tight junctions; HDAC inhibition → promote neurogenesis	Human + Animal + *In vitro* (31–33)
Propionate	74.08	Commensal fermentation of dietary fiber	MCT mediated active transport	Block HK2/IDO1 expression; Stabilize BBB structure	Human + Animal + *In vitro* (31–33)
Butyrate	88.11	Specific metabolite of Bifidobacterium, Faecalibacterium	MCT mediated active transport	Potent HDAC inhibitor; Enhance astrocytic antioxidant capacity; Reduce Aβ deposition	Human + Animal + *In vitro* (31, 33, 34)
Microbiota-derived GABA	103.12	Synthesized by Lactobacillus, Bifidobacterium	Passive diffusion + MCT (GAT-2/SLC6A12)	Antagonize QA-induced excitotoxicity; Modulate inhibitory neural circuits	Animal + *In vitro* (35, 36)
Indole-3-propionic Acid	175.19	Tryptophan fermentation by probiotic Clostridium	Passive diffusion	AhR ligand → Treg↑/Th17↓; Preemptively intercept IDO runaway	Human + Animal + *In vitro* (37–39)

*Evidence Level: Human/Clinical = evidence derived from human/clinical studies (e.g., patient cohorts, CSF/serum metabolomics); Animal = in vivo animal models; In vitro = cell-culture experiments. ‘+’ indicates combined evidence across multiple levels.

**Table 3:** Systemic host metabolic intermediates.

Molecule	MW(Da)	Generation source	BBB crossing mechanism	Central pathogenic/protective mechanism	Evidence level
Secondary Bile Acids	360–490	Hepatic primary bile acids modified by gut microbiota	Passive diffusion	Activate FXR/TGR5; Regulate cholesterol transport; Stabilize BBB	Human + Animal + *In vitro* (39–41)
Hydrophobic Bile Acids	380–495	High-fat diet/Liver metabolic disorder induced dysbiosis	Passive diffusion	Damage endothelial mitochondria; Degrade ZO-1/Occludin	Human + Animal + *In vitro* (40–42)
Trimethylamine N-oxide	75.11	Choline/TMA (gut) → TMAO (Liver FMO3)	Passive diffusion	Induce endothelial apoptosis; Inhibit autophagy → impair Aβ clearance	Human + Animal + *In vitro* (43–45)
Advanced Glycation End-products	Mixed	Hepatic non-enzymatic glycation	Passive diffusion	RAGE binding → NF-κB activation; Promote Aβ cross-linking	Human + Animal + *In vitro* (38, 46)

No metabolite currently has a dedicated systematic review or meta-analysis in AD; existing human evidence derives from primary cohort and cross-sectional studies, and several supporting citations are narrative reviews. A formal evidence synthesis remains a stated gap.

### 3.1 Peripheral immune-derived metabolic small molecules: drivers and counterbalances in early AD pathogenesis

**Figure 2:**
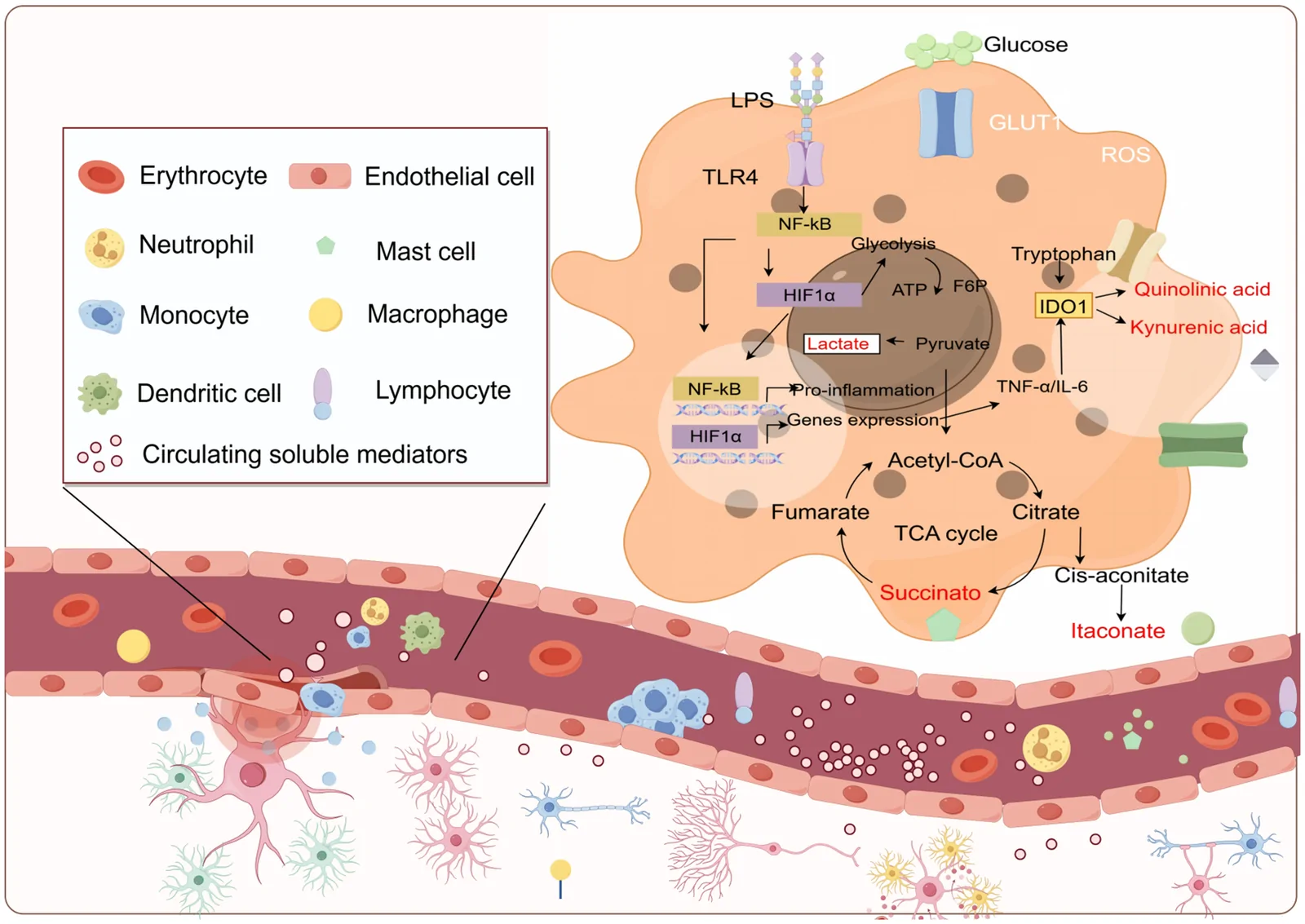
Generation and BBB penetration of soluble small-molecule metabolites from metabolically reprogrammed peripheral immune cells. Metabolic reprogramming triggered by systemic inflammation reshapes tricarboxylic acid (TCA) cycle metabolism in monocytes, macrophages and lymphocytes, producing multiple low-molecular-weight metabolites that readily permeate the physiological intact BBB to initiate early neuroinflammation. TCA, tricarboxylic acid; LPS, lipopolysaccharide; ROS, reactive oxygen species. Created by figdraw.com.

Peripheral immune-derived metabolites constitute the first of these three categories and are considered first because their release is temporally coupled to the low-grade systemic inflammation that precedes clinically overt Alzheimer’s disease. The subsection below surveys the small molecules most consistently implicated as candidate early mediators of clinically silent, preclinical neuroinflammation—from canonical glycolytic and tricarboxylic-acid-cycle intermediates to tryptophan-derived species—and evaluates the transport routes and intracellular cascades through which they may act before structural barrier breakdown (**Figure 2**).

#### 3.1.1 Lactate

Peripherally derived lactate is functionally and temporally distinct from brain-intrinsic lactate produced via the astrocyte-neuron lactate shuttle (ANLS). Under physiological conditions, endogenous cerebral lactate solely supports synaptic energy metabolism, with significant cerebral lactate elevation only observed in late-stage AD (47).

Aging-triggered inflammation upregulates lactate dehydrogenase A (LDHA) via Toll-like receptor 4 (TLR4)/nuclear factor kappa B (NF-κB) and HIF-1α signaling, elevating peripheral lactate secretion by approximately 3–5 fold (48). Circulating lactate penetrates the BBB through endothelial monocarboxylate transporters (MCTs) MCT1/MCT4 and neuronal MCT2, accumulating in vulnerable brain regions including the hippocampus and posterior cingulate gyrus. Cerebrospinal fluid (CSF) metabolomics and magnetic resonance spectroscopy (MRS) data confirm that elevated lactate in prodromal AD originates predominantly from peripheral circulation rather than local CNS synthesis (20).

Within brain parenchyma, lactate acts as an epigenetic signal donor to induce histone lactylation (H4K12la, H3K18la) and co-activate NF-κB/p65 via p300/CBP coactivators. This cascade polarizes microglia toward a pro-inflammatory M1 phenotype, upregulates glycolytic PKM2, and sustains open chromatin conformation at pro-inflammatory gene loci, converting transient peripheral immune stimuli into persistent chronic neuroinflammation—a molecular signature distinguishing physiological brain aging from pathological AD progression (18).

Concurrently, lactate mediates non-covalent lactylation of APP K612 and tau K331, enhances BACE1 activity to generate toxic Aβ oligomers, and accelerates tau hyperphosphorylation at Ser396/Ser404 through GSK-3β activation, simultaneously triggering two independent neuronal toxic cascades (49). Owing to its prominent role in contributing to preclinical pathological remodeling, CSF lactate represents a promising non-invasive biomarker for prodromal AD. Notably, the disease-driving role of histone lactylation in human AD progression is inferred mainly from rodent and cell-culture studies; its contribution to clinical AD pathogenesis remains to be established in human brain tissue (**Table 1**).

#### 3.1.2 Succinate

Succinate accumulates intracellularly in activated immune cells prior to systemic release, driven by tricarboxylic acid (TCA) cycle stalling and succinate dehydrogenase (SDH)-mediated reverse electron transfer (RET), supporting its recognition as a canonical immunometabolic hallmark (50).

With a molecular weight of 118 Da and moderate lipophilicity, succinate bypasses the size and polarity filtration of intact BBB, binds succinate receptor 1 (SUCNR1) to activate HIF-1α/ROS cascades in microglia and neurons, inhibits mitochondrial Complex I/III, and disrupts intracellular redox homeostasis (22). Recent work refutes the oversimplified linear model positing succinate accumulation suppresses SDH: Sirt5 deletion induces site-specific succinylation of the SDH catalytic subunit (SDHA), enhancing SDH catalytic activity and confirming that ROS overproduction is driven by the SDH-RET axis rather than simple SDH inactivation.

Pathologically, the succinate-ROS axis activates GSK-3β to accelerate tau hyperphosphorylation and neurofibrillary tangle formation, while exacerbating glial inflammatory activation and synaptic loss (21). Clinical cohort data suggest that plasma and CSF succinate concentrations negatively correlate with brain tau PET load and cognitive performance in mild cognitive impairment (MCI) patients, supporting its utility as an ultra-early diagnostic biomarker.

Pharmacological α-ketoglutarate dehydrogenase (α-KGDH) inhibition via succinyl phosphonate blocks the succinylation-SDH-ROS axis, alleviates microglial senescence *in vitro*, and mitigates neuroinflammation *in vivo*, supporting its candidacy as a viable therapeutic target (51).

#### 3.1.3 Tryptophan-derived kynurenine

Peripheral immune activation upregulates indoleamine 2,3-dioxygenase 1 (IDO1), which catalyzes tryptophan degradation into kynurenine (KYN)—the sole tryptophan metabolite capable of efficient trans-BBB transport via L-type amino acid transporter 1 (LAT1) (26). Circulating KYN readily traverses the BBB and undergoes secondary metabolism by resident microglia and astrocytes to yield neurotoxic quinolinic acid (QA) and neuroprotective kynurenic acid (KYNA); notably, peripherally derived QA and KYNA cannot independently cross the intact BBB (27). Excessive intracerebral QA drives persistent NMDA receptor overactivation, intracellular calcium overload, and irreversible neuronal excitotoxicity, whereas KYNA counteracts this excitatory damage to maintain neuronal homeostasis. Chronic systemic inflammation sustains elevated circulating KYN, biases cerebral tryptophan metabolism toward QA overproduction, and elevates the QA/KYNA ratio—a sensitive proxy for early prodromal AD pathology (25).

#### 3.1.4 Other minor peripheral immune-derived immunometabolites

Beyond the three core BBB-permeable metabolites above, activated monocytes, macrophages, and T lymphocytes generate auxiliary circulating immunometabolites that traverse the intact BBB via dedicated transporters. α-ketoglutarate (α-KG) and citrate—two abundant TCA cycle intermediates that accumulate during immune glycolytic reprogramming—enter the CNS via organic anion transporters with consistent efficiency (30). Upon brain entry, both metabolites remodel the microglial epigenetic landscape, enhance mitochondrial ROS production, and synergistically amplify lactate- and succinate-driven pro-inflammatory signaling, accelerating tau hyperphosphorylation and synaptic degeneration (29). In contrast, native itaconate has negligible BBB permeability due to high polarity, with only trace levels detected in brain parenchyma under physiological or prodromal AD conditions. Consequently, it cannot counterbalance dominant pro-inflammatory metabolites and exerts minimal regulatory effects in early disease stages (23).

### 3.2 Gut microbiota-derived metabolic small molecules: core counter-regulatory mediators of inflammatory homeostasis

**Figure 3:**
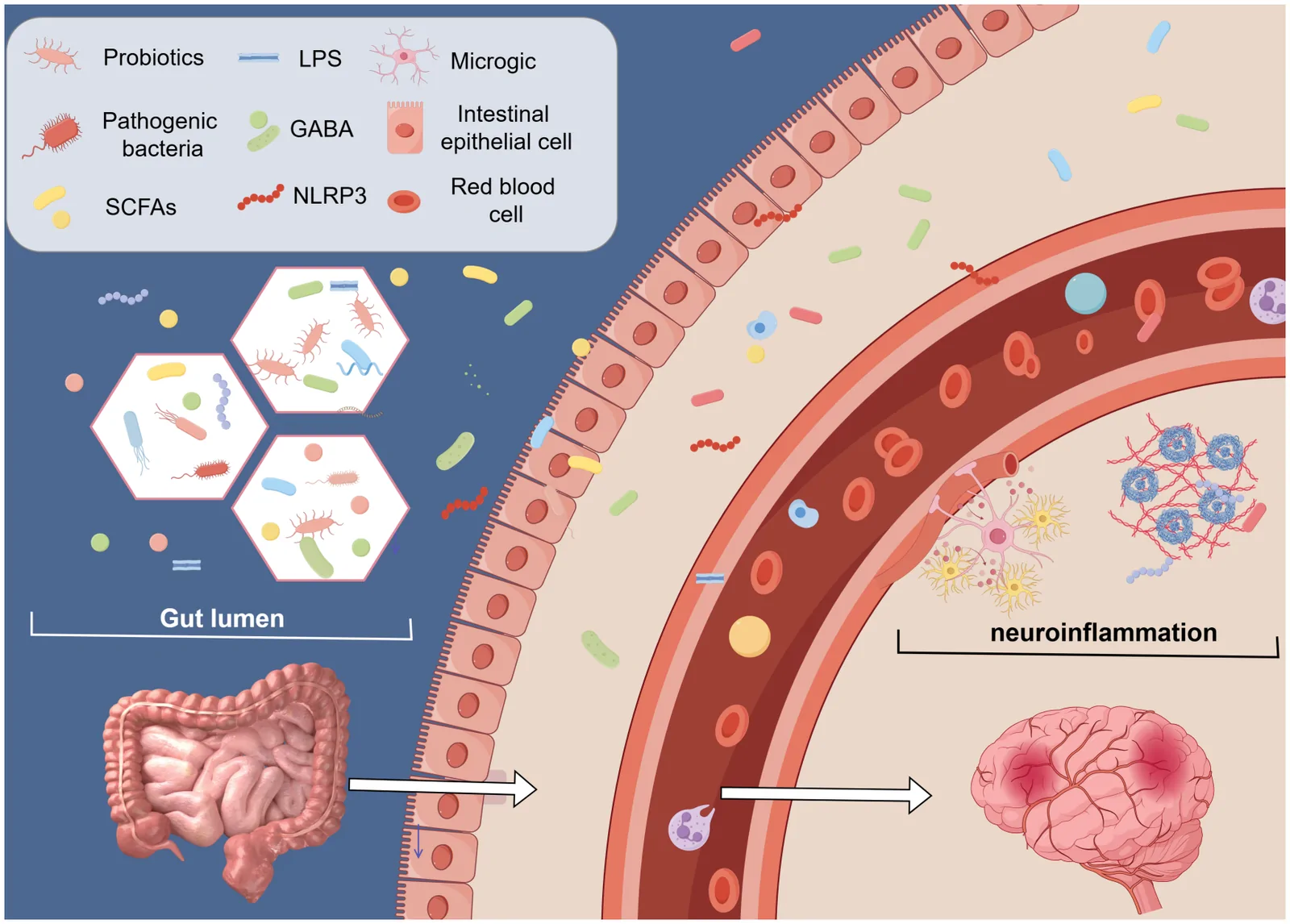
Gut microbiota-derived metabolites mediate peripheral-central immunometabolic crosstalk in AD. Short-chain fatty acids (SCFAs), microbiota-produced GABA and indole derivatives generated by intestinal commensal bacteria enter systemic circulation, cross the BBB and exert bidirectional regulatory effects on central neuroinflammation. Gut dysbiosis disrupts this protective metabolic network and accelerates AD pathological progression. Created by figdraw.com.

Distinct from pro-inflammatory peripheral immune-derived metabolites, gut microbiota-derived metabolites constitute a dedicated counter-regulatory axis that buffers central nervous system (CNS) from excessive peripheral insults, primarily via metabolic anti-inflammation and neuroexcitatory modulation. Three core subgroups with validated CNS-penetrating capacity are elaborated below (**Figure 3**).

#### 3.2.1 Short-chain fatty acids

Acetate, propionate and butyrate are the main bioactive metabolites fermented by intestinal commensal bacteria. Although they share core pharmacological activities including HDAC inhibition and intestinal mucosal barrier maintenance, their functions are differentiated *in vivo* (52).

Acetate and propionate exert mainly peripheral regulatory effects. Through HDAC inhibition, they suppress the pro-inflammatory enzymes HK2 and IDO1 and block the excessive production of lactate and neurotoxic kynurenine derivatives. They also limit intestinal LPS translocation, thereby relieving systemic low-grade inflammation.

Acetate and propionate can cross the BBB via monocarboxylate transporters, but their accumulation in brain tissue is far lower than that of butyrate, so they exert only mild auxiliary protective effects in the CNS (33).

Among the three SCFAs, butyrate possesses the strongest BBB permeability relying on monocarboxylate transporters, acting as the primary microbial signal to directly regulate CNS homeostasis. After entering brain parenchyma, butyrate activates cerebral AMPK-SIRT1 cascade to inhibit excessive activation of microglial NLRP3 inflammasome, relieve neuronal metabolic dysfunction, and counteract tau hyperphosphorylation induced by peripheral immunometabolites (53).

Multiple studies have confirmed that circulating butyrate levels decrease significantly in mild AD patients, and its concentration is positively correlated with cognitive function while negatively correlated with cerebral tau deposition (32).

#### 3.2.2 Microbiota-derived GABA

Microbially synthesized γ-aminobutyric acid (GABA) complements the functional deficiency of SCFAs in antagonizing glutamate excitotoxicity, jointly constructing a dual regulatory system integrating metabolic anti-inflammation and neuroprotection (54). After being synthesized by beneficial intestinal bacteria and released into circulation, GABA crosses intact BBB via passive diffusion and MCT-mediated transport, selectively binds neuronal GABA_a_ receptors, and competitively counteracts NMDA receptor overactivation induced by quinolinic acid, thereby alleviating intracellular calcium overload and synaptic damage (36). During AD progression, pathogenic strains such as Escherichia coli overgrow and displace GABA-producing commensal bacteria, reducing peripheral and central GABA levels and breaking the excitatory-inhibitory balance of neurons (55). Different from endogenously synthesized brain GABA, microbiota-derived GABA provides an actionable target for dietary intervention and probiotic therapy (35).

#### 3.2.3 Microbiota-derived indoles

Indole derivatives including indole-3-propionic acid (IPA) and indole-3-aldehyde (IALD) are tryptophan fermentation products of intestinal commensal bacteria, which antagonize the pro-inflammatory IDO1-kynurenine pathway (37). As endogenous agonists of aryl hydrocarbon receptor (AhR), these indole metabolites facilitate regulatory T cell (Treg) differentiation and suppress Th17 polarization, indirectly limiting excessive host IDO1 activation and reducing the production of neurotoxic quinolinic acid. Distinct from circulating kynurenine derived from peripheral immune cells, microbial indoles mediate immune homeostasis through the indole–AhR–Treg signaling axis (56). Multiple studies confirm that gut dysbiosis in AD patients reduces intestinal indole content and elevates systemic IDO activity. Insufficient indole supply leads to persistent overactivation of IDO1 and imbalance of QA/KYNA ratio, which can be used as an early marker of impaired anti-inflammatory compensation.

### 3.3 Systemic host metabolic intermediates

Distinct from peripheral immune-derived metabolites that have been linked to the initiation of preclinical AD lesions and gut microbiota-derived metabolites that function as homeostatic balancers, systemic host metabolic intermediates serve as secondary amplifiers that amplify established inflammation during mid-to-late AD.

#### 3.3.1 Advanced glycation end-products

AGEs are heterogeneous metabolic adducts generated via non-enzymatic glycation between reducing sugars and tissue proteins or lipids, with the liver dominating circulating AGE clearance and accumulation (38). Aging, persistent hyperglycemia, and high-fat diets accelerate systemic AGE synthesis and elevate peripheral concentrations. Small-molecule monomeric AGEs penetrate the structurally intact BBB via passive lipid diffusion, whereas large protein-bound AGE complexes only leak into brain parenchyma after irreversible tight junction degradation in mid-to-late AD (46).

Physiological low-concentration AGEs participate only in mild vascular signaling without neurotoxicity. In contrast, excessive AGE accumulation binds endothelial RAGE receptors to activate downstream NF-κB signaling and induce oxidative stress, further degrading cerebrovascular tight junctions and impairing BBB selectivity. Concurrently, AGEs promote abnormal cross-linking and aggregation of cerebral Aβ peptides and synergistically amplify M1 microglial polarization induced by peripheral immune-derived lactate and succinate, jointly accelerating tau hyperphosphorylation and irreversible synaptic damage in AD lesions (57).

#### 3.3.2 Bile acid derivatives

Primary bile acids are primarily synthesized in hepatocytes, while secondary bile acids (e.g., deoxycholic acid and ursodeoxycholic acid, 350–400 Da) are biotransformed from primary substrates via intestinal commensal microbiota (58). Their amphipathic properties enable bile acid derivatives to penetrate the anatomically intact BBB via passive diffusion, independent of solute carrier transporters (40).

Under physiological conditions, protective hydrophilic bile acids activate endothelial FXR/TGR5 signaling to stabilize mitochondrial function, relieve oxidative stress, and maintain neurovascular unit (NVU) tight junction integrity.

However, combined hepatic metabolic disorder and gut dysbiosis in AD significantly elevate circulating cytotoxic hydrophobic bile acids. These target endothelial mitochondria to trigger mitochondrial permeability transition pore opening and downregulate tight junction proteins (ZO-1, occludin), ultimately destroying BBB integrity (39).

Increased cerebrovascular permeability permits massive infiltration of peripheral pro-inflammatory metabolites, forming a positive pathological loop that aggravates Aβ plaque deposition and neurofibrillary tangle formation—supporting the characterization of bile acid derivatives as key secondary amplifiers of AD central pathology.

#### 3.3.3 Trimethylamine N-oxide

TMAO (75 Da) is a prototypical gut-liver axis metabolite reflecting systemic lipid disturbance: intestinal microbiota convert dietary choline and betaine into trimethylamine, which is hepatically oxidized into TMAO via flavin-containing monooxygenase 3 (FMO3) (45).

TMAO traverses the intact BBB via organic cation transporters (OCTs) on brain capillary endothelial cells (44). Clinical cohorts confirm markedly elevated circulating TMAO in MCI and late AD, with higher levels correlating positively with AD risk and accelerated cognitive decline (59).

Mechanistically, accumulated TMAO induces endothelial oxidative stress and apoptosis, directly damaging BBB integrity. Upon central entry, TMAO drives microglial metabolic reprogramming toward aerobic glycolysis and M1 polarization, activates the NLRP3 inflammasome cascade to amplify neuroinflammation, and concurrently inhibits glial-neuronal autophagy flux—hindering Aβ clearance and accelerating plaque and tau tangle deposition.

Consistent with its role as a secondary amplifier, TMAO cannot independently induce asymptomatic preclinical neuroinflammation but significantly exacerbates central pathology initiated by peripheral immune-derived metabolites once mild barrier dysfunction and microglial activation occur (45).

#### 3.4 Cross-category overlaps and an integrative perspective

In practice, the three-category division is heuristic, and several circulating metabolites span more than one biosynthetic origin or regulatory pathway, blurring the categorical boundaries. Trimethylamine N-oxide (TMAO), for example, is generated from host-derived trimethylamine that is oxidized by gut microbiota, whereas succinate is produced by both activated immune cells and gut microbes; both therefore reside at the interface of categories rather than within a single strict class. Such overlaps have been acknowledged where relevant in the preceding subsections. Taken together, the evidence compiled in **Tables 1**-**3** indicates that most core connections are presently supported mainly by preclinical *in vitro* and rodent data and await confirmation in large human AD cohorts, although several individual metabolite biomarkers already show consistent clinical association. A complete account of peripheral–central immunometabolic crosstalk ultimately requires integrating signals across categories rather than treating them in isolation.

## 4 Trans-barrier transport mechanisms and regulatory roles of metabolic small molecules

### 4.1 Carrier-mediated active transport: the gateway of preclinical initiation

We posit that during prodromal AD with anatomically intact tight junctions, ATP-dependent carrier-mediated active transport may act as the primary early conduit for circulating small metabolites to cross the BBB and trigger subclinical neuroinflammation. This stage-dependent transport hypothesis is predominantly supported by *in vitro* and rodent experiments and awaits validation in human brain tissues. This system comprises two opposing families: solute carrier (SLC) influx transporters and ATP-binding cassette (ABC) efflux pumps, which collectively maintain central metabolic homeostasis. Substrate selectivity is isoform-specific: MCT1/MCT4 mediate monocarboxylates (e.g., lactate, SCFAs); LAT1/2 facilitate bidirectional exchange of tryptophan-derived kynurenine; and OAT/OATP families transport dicarboxylic acids such as succinate and bile acid derivatives (60).

**Figure 4:**
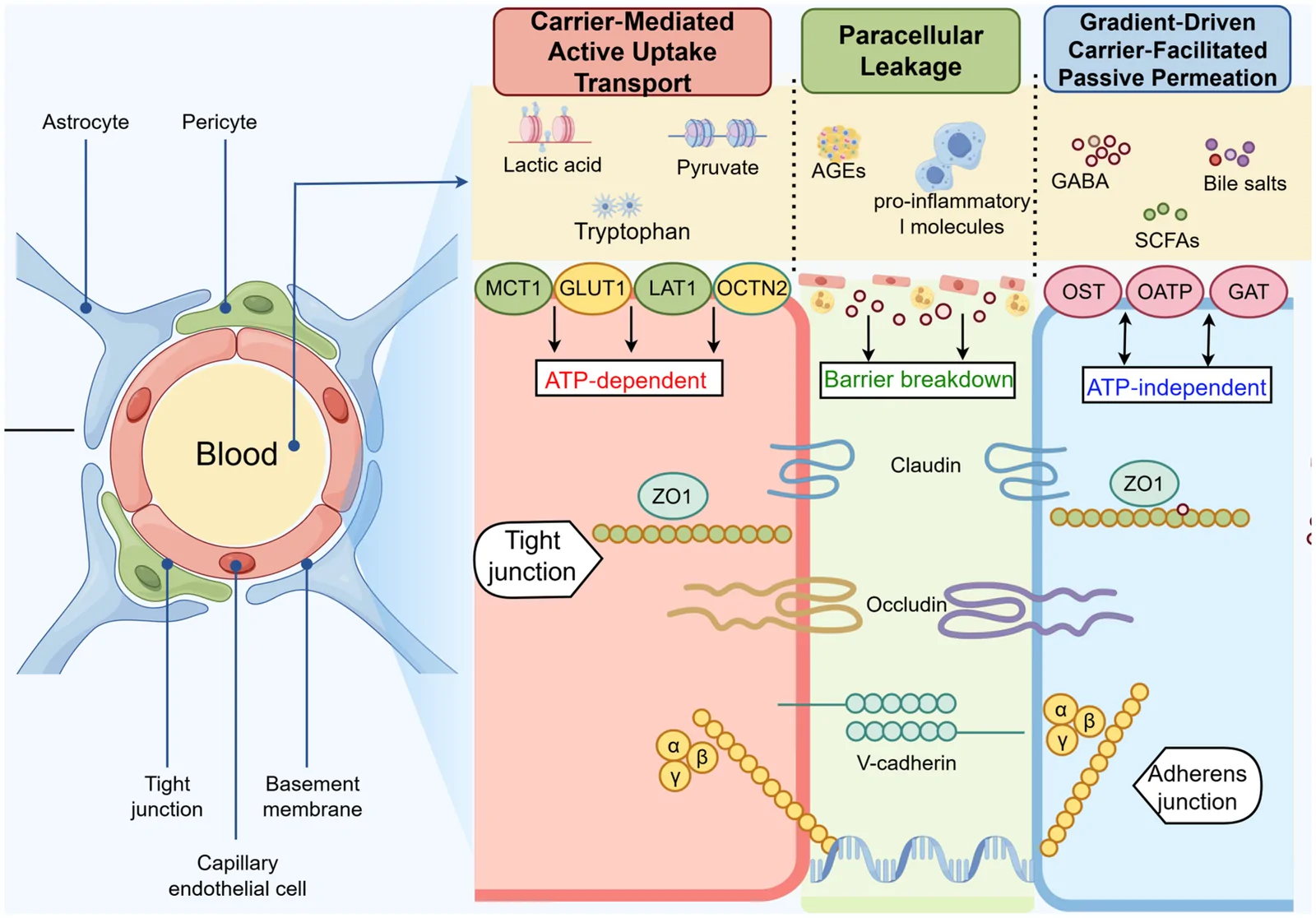
Three distinct transport mechanisms of circulating metabolic small molecules across the BBB. Three transport modes govern the entry of circulating metabolites into the brain parenchyma at different AD stages: ATP-dependent carrier-mediated active transport, gradient-driven facilitated diffusion, and non-selective paracellular leakage triggered by irreversible tight junction damage. Core transporters, tight junction proteins and representative permeable metabolites are labeled in the diagram. Created by figdraw.com.

Chronic systemic low-grade inflammation in aging engages TLR4/NF-κB and HIF-1α signaling, transcriptionally upregulating SLC influx carriers while concurrently suppressing ABCG2 and ABCC1 efflux pumps. This aberrant transporter gradient impedes metabolite clearance from the brain parenchyma, promoting the cerebral accumulation of toxic immunometabolites—a core molecular basis for early neuroinflammation preceding tight junction rupture. The unchecked influx of lactate, succinate, and kynurenine further drives microglial M1 polarization and NLRP3 inflammasome priming, laying the pathogenic groundwork for subsequent AD progression (**Figure 4**).

### 4.2 Facilitated diffusion: auxiliary modulation across disease continuum

Facilitated diffusion is defined as ATP-independent transmembrane transport driven by concentration gradients, mediated by low-affinity carrier proteins, and applicable principally to amphipathic small molecules with a log P between 1.5 and 2.5 (61). Distinct from energy-dependent active transport, this pathway requires no ATP hydrolysis and exhibits broader substrate permissivity, functioning as a supplementary route throughout the AD continuum. Representative carriers include GAT-2 for microbiota-derived GABA and OATP subtypes for lipophilic bile acids (62).

Under physiological homeostasis, favorable concentration gradients drive the central penetration of protective microbial metabolites (e.g., butyrate, GABA), activating cerebral AMPK-SIRT1 signaling to constrain microglial overactivation. As AD progresses and dysbiosis ensues, circulating levels of protective metabolites plummet while toxic host-derived metabolites (e.g., TMAO) accumulate, reversing the gradient to amplify central oxidative injury synergistically with active transport. Unlike active transport, which initiates early lesions, facilitated diffusion exerts secondary regulatory effects—modulating neuroinflammation intensity based on peripheral fluctuations—thereby bridging early initiation and late amplification.

### 4.3 Barrier-collapse paracellular flux: late-stage pathological amplification

Paracellular leakage is a pathological transport mode largely confined to mid-to-late AD, incapable of independently triggering preclinical neuroinflammation behind an intact BBB. This pathway arises from the irreversible degradation of tight junction proteins (ZO-1, occludin, claudin-5), a process driven by chronic Aβ deposition and persistent glial activation independent of transporter regulation​ (63). Endothelial intercellular gaps eliminate the BBB’s size and polarity selectivity, permitting unrestricted infiltration.

In this collapsed state, macromolecular cytokines (IL-6, TNF-α), protein-bound AGEs, and hydrophobic bile acids infiltrate freely alongside massive immune cell extravasation (42). Unchecked paracellular flux has been proposed to drive a self-amplifying feedback loop: metastatic infiltration exacerbates glial activation, accelerating tight junction proteolysis and converting reversible metabolic disturbances into irreversible neurodegeneration (41). Thus, this pathway functions as the final common pathway​ for amplifying all peripheral pathogenic signals rather than an independent initiator of early disease.

## 5 Temporally layered intracellular signaling networks regulating neuroinflammation via brain-infiltrating metabolites

Circulating bioactive small molecules breach the blood-brain barrier to access brain parenchyma, triggering highly ordered, stage-specific intracellular signaling cascades rather than stochastic cellular disruption. Five interlinked core modules are proposed to be sequentially activated across the Alzheimer’s disease (AD) continuum—from the prodromal phase to advanced dementia—collectively dictating the functional state of microglia, astrocytes, and neurons. Reciprocal crosstalk between these modules determines whether peripheral metabolic stimulation evolves into transient cellular stress or sustained chronic neuroinflammation (64). This stage-dependent ordering is a heuristic framework advanced by this review and awaits confirmation by longitudinal human staging studies.

### 5.1 Mitochondrial redox homeostasis pathway

Mitochondria serve as the primary upstream sensor for infiltrating metabolites, with activation peaking during prodromal AD. Peripheral immune-derived lactate and succinate target the inner mitochondrial membrane, disrupting electron transport chain Complex I/III to induce robust ROS bursts; succinate further exacerbates oxidative injury via SDH-dependent reverse electron transfer (65). Concurrently, host-derived TMAO and AGEs synergistically open mitochondrial permeability transition pores, amplifying oxidative damage (66). In contrast, gut microbiota-derived butyrate and itaconate stabilize mitochondrial architecture and suppress SDH-mediated ROS generation, mitigating early microglial lesions (67). As the initiating trigger for all downstream cascades, mitochondrial redox imbalance directly perturbs cellular nutrient sensing, as elaborated below.

### 5.2 AMPK-SIRT1-mTOR nutrient-sensing axis

Mitochondrial ROS accumulation suppresses AMPK activity and downregulates SIRT1 expression, culminating in mTORC1 hyperactivation—a hallmark of pathological remodeling during mild cognitive impairment (MCI) (68). Hyperactive mTOR drives microglial glycolytic reprogramming and M1 polarization. Conversely, gut-derived SCFAs and microbial GABA reverse this cascade, restoring the M2 anti-inflammatory phenotype via AMPK activation. A closed feedback loop tightly couples mitochondrial function to this nutrient-sensing axis; critically, persistent mTOR overactivation establishes the prerequisite for subsequent NLRP3 inflammasome assembly (69).

### 5.3 NLRP3 inflammasome pathway

Sustained mTOR hyperactivation and mitochondrial oxidative stress converge to drive NLRP3 oligomerization and caspase-1 activation, marking the pathological transition from compensated MCI to progressive AD (24). Activated NLRP3 facilitates IL-1β and IL-18 maturation, instigating overt neuroinflammation (70). While itaconate inhibits NLRP3 priming via Nrf2 activation, SCFAs downregulate NLRP3 transcription, dampening glial inflammatory responsiveness (71). Robust inflammasome activation is largely absent during the prodromal stage when the NVU remains intact. Prolonged activation induces stable glycolipid remodeling within glial cells, bridging innate immune activation to metabolic dysfunction (72).

### 5.4 Glycolipid metabolic reprogramming pathway

Prolonged NLRP3 signaling locks microglia into a persistent glycolytic shift—a defining feature of mid-to-late AD (73). Peripheral immune lactate and succinate continuously upregulate glycolytic enzymes (e.g., PKM2), while hepatic AGEs and TMAO trigger lipid peroxidation and aberrant lipid droplet accumulation (74). This remodeled metabolic phenotype sustains M1 polarization independent of ongoing peripheral stimuli and synergistically exacerbates NVU leakage and cerebral Aβ/tau pathology (75). This sustained glycolipid disorder has been proposed to provide the metabolic foundation for relatively stable epigenetic immune memory; whether such immune memory is truly permanent or reversible in humans remains unresolved and is inferred primarily from preclinical studies (Evidence Level: Animal + *In vitro*).

### 5.5 Histone reversible epigenetic modification pathway

Histone modification has been proposed to represent the terminal regulatory layer cementing chronic inflammatory memory, a hallmark of late-stage AD. Sustained cerebral lactate of peripheral origin acts as a lactylation donor, inducing H4K12la and H3K18la. These modifications maintain open chromatin conformation at pro-inflammatory gene loci, rendering glia hyper-responsive even after peripheral stimuli cease. This epigenetic reprogramming distinguishes AD neurodegeneration from physiological aging and defines a critical therapeutic window for early immunometabolic intervention.

Our synthesis of scattered preclinical studies suggests that these five interconnected signaling modules may form a self-amplifying pathological vicious cycle across the AD disease spectrum. This sequential cascade model has not yet been confirmed via human longitudinal cohort analysis. Circulating metabolites breach the NVU to sequentially activate these intracellular cascades. In turn, persistent glial activation degrades tight junction proteins and dysregulates solute carrier influx transporters, further promoting the cerebral accumulation of toxic metabolites. This closed-loop vicious cycle perpetuates pathology across all AD stages.

## 6 Limitations and future research perspectives

### 6.1 Critical appraisal of conflicting and negative evidence

While the framework advanced herein emphasizes the pathogenic contribution of circulating metabolites, the literature is not uniformly confirmatory. Several human metabolomics and cohort studies report inconsistent or null associations for individual metabolites—for example, heterogeneous direction and magnitude of TMAO and SCFA changes across AD cohorts—and most are cross-sectional, leaving the direction of causality (metabolites driving versus passively reflecting neurodegeneration) unresolved (76). Certain metabolites are also context-dependent: the kynurenine pathway bifurcates into neurotoxic (quinolinic acid) and neuroprotective (kynurenic acid) branches (25), whereas butyrate shows protective actions in some models yet variable outcomes in human interventional trials (34).

Crucially, the bulk of mechanistic evidence derives from monogenic transgenic mouse models, cultured cell systems, and cross-sectional metabolomics rather than prospective human cohorts (76). *In vivo* validation by human neuroinflammation PET imaging is essentially absent (77), circulating metabolites have not been validated as diagnostic or therapeutic targets in interventional human studies, and reproducibility across populations differing in APOE status, sex, age, diet, and ancestry remains largely untested. Longitudinal human validation is therefore essential before the proposed axis can be regarded as established.

### 6.2 Proposed conceptual framework and current evidence gaps

As comprehensively catalogued in **Tables 1**-**3**, the experimental support behind the proposed axis is uneven in maturity. Several metabolite classes — short-chain fatty acids (e.g., acetate, propionate, butyrate), bile acids, trimethylamine N-oxide, kynurenate and α-ketoglutarate — already carry human or clinical-cohort evidence, whereas lactate, succinate and other predominantly preclinical metabolites remain established chiefly in cell-culture and animal models; the transmembrane BBB routes and the downstream intracellular cascades, in particular, still rest largely on rodent and *in vitro* work. Consequently, the axis is best read in a maturity-graded manner: individual molecular events are better established than the integrated transport-to-cascade sequence, and the tripartite classification should be regarded as an organizational heuristic rather than settled consensus biology.

Resolving this graded evidential map is central to clinical translation. Looking ahead, prioritizing candidate metabolites by their evidence maturity and profiling them within APOE- and comorbidity-stratified longitudinal human cohorts (78) — coupled with *in vivo* neuroinflammation PET (79) — should help consolidate the present framework into testable, population-specific panels of early fluid biomarkers and stage-adapted peripheral immunometabolic interventions (19, 80).

### 6.3 AD heterogeneity and the multifactorial roles of circulating metabolites

AD is a profoundly heterogeneous disorder shaped by genetic, vascular, metabolic, inflammatory, sex-related, age-related, and environmental determinants, so the peripheral-central axis cannot be assumed to act as a single uniform mechanism across patients. Its relative weight probably shifts with disease stage — from prodromal carrier transport and subclinical neuroinflammation to late-stage barrier collapse — and with patient subgroup (81, 82). Individual metabolites are likewise multifunctional within this axis: depending on concentration, timing, and cellular context, the same molecule may serve as a causal driver (e.g., sustained lactate or succinate fueling NLRP3 activation), an epigenetic modifier (lactate as a lactylation donor), a biomarker (plasma/CSF TMAO and SCFAs; 31, 45), or a downstream consequence of neurodegeneration, and these roles are not mutually exclusive.

Appreciating this context-dependence reframes the axis as a stratified, rather than binary, model of risk. Future stratified, longitudinal human studies — capturing how a given metabolite’s contribution shifts with APOE status, comorbidity, and disease stage — will be essential to refine this view and to guide precision prevention and stage-tailored immunometabolic strategies.

## 7 Conclusion

In summary, this review delineates the peripheral-central immunometabolic axis that operates in preclinical Alzheimer’s disease (AD) before overt blood-brain barrier (BBB) disruption. We organize circulating metabolites into three heuristic groups by biosynthetic origin — peripheral immune-derived, gut microbiota-derived, and systemic host metabolic intermediates — and trace how, through stage-dependent trans-BBB transport, they may activate intracellular cascades that sustain chronic neuroinflammation and epigenetic immune memory. In contrast to classical models emphasizing late-stage barrier rupture, this perspective positions immunometabolic crosstalk as an early, peripherally rooted contributor to silent prodromal neuroinflammation, and thereby provides a mechanistic basis for ultra-early fluid biomarkers and peripheral disease-modifying therapies that can help orient future basic and translational research. We note, however, that the proposed axis remains a heuristic synthesis: although several individual metabolites already show consistent clinical association, the integrated transport-to-cascade sequence still requires validation in large human cohorts. By clarifying this multi-stage axis, the framework may help prioritize peripheral metabolic targets, staging biomarkers, and intervention time windows for subsequent human studies.
